# Enucleation-Induced Rat Adrenal Gland Regeneration: Expression Profile of Selected Genes Involved in Control of Adrenocortical Cell Proliferation

**DOI:** 10.1155/2014/130359

**Published:** 2014-11-06

**Authors:** Marianna Tyczewska, Marcin Rucinski, Agnieszka Ziolkowska, Marta Szyszka, Marcin Trejter, Anna Hochol-Molenda, Krzysztof W. Nowak, Ludwik K. Malendowicz

**Affiliations:** ^1^Department of Histology and Embryology, Poznan University of Medical Sciences, Poznan, Poland; ^2^Department of Animal Physiology and Biochemistry, Poznan University of Life Sciences, Poznan, Poland

## Abstract

Enucleation-induced adrenal regeneration is a highly controlled process; however, only some elements involved in this process have been recognized. Therefore, we performed studies on regenerating rat adrenals. Microarray RNA analysis and QPCR revealed that enucleation resulted in a rapid elevation of expression of genes involved in response to wounding, defense response, and in immunological processes. Factors encoded by these genes obscure possible priming effects of various cytokines on initiation of regeneration. In regenerating adrenals we identified over 100 up- or downregulated genes involved in adrenocortical cell proliferation. The changes were most significant at days 2-3 after enucleation and their number decreased during regeneration. For example, expression analysis revealed a notable upregulation of the growth arrest gene, Gadd45, only 24 hours after surgery while expression of cyclin B1 and Cdk1 genes was notably elevated between days 1–8 of regeneration. These changes were accompanied by changes in expression levels of numerous growth factors and immediate-early transcription factors genes. Despite notable differences in mechanisms of adrenal and liver regeneration, in regenerating adrenals we identified genes, the expression of which is well recognized in regenerating liver. Thus, it seems legitimate to suggest that, in the rat, the general model of liver and adrenal regeneration demonstrate some degree of similarity.

## 1. Introduction 

Enucleation-induced adrenal regeneration—one of the types of adrenal gland growth* in vivo*—depends on adrenocortical cell hyperplasia and hypertrophy and is connected with differentiation of parenchymal cells [1, 2]. Removal of both cortex and medulla triggers rapid adrenocortical growth, leading within 3-4 weeks to restoration of adrenocortical structure and function [3–10]. Enucleation-evoked rapid decrease of corticosteroid concentrations in blood leads to compensatory hypersecretion of ACTH and N-proopiomelanocortin peptides (N-POMC), which then stimulate regeneration process [11–13]. Regeneration of adrenals starts from progenitor and some zona glomerulosa cells, left attached to the connective tissue capsule [3, 14–16]. The rate and magnitude of changes in the regenerating rat adrenal cortex are associated primarily with the reconstruction of the zona fasciculata, the structure of which is formed even before day 20 of regeneration. On the other hand, restoration of the zona glomerulosa before day 30 of regeneration remains incomplete [16]. Enucleation-induced adrenal regeneration is a highly controlled process; however, only some elements involved in this process have been recognized.

The first three days after adrenal enucleation combine clot organization and preparation for cell division (initial or early phase) and from the end of day 3 proliferation phase starts, which persists to day 7 of gland regeneration [2]. On basis of the adrenal weight and DNA content it is assumed that the regeneration is complete by week 3 after enucleation [1]. When compared with the widely studied partial hepatectomy-induced (removal about 75% of the liver) liver regeneration, the time span of adrenal regeneration is notably longer. Hepatocyte DNA replication starts at 12 h after surgery in rats, and the first wave of DNA synthesis occurs at about 24 h [17]. The entire process of liver regeneration in the rat is commonly divided into the following phases: (i) immediate early phase (2 h); (ii) early phase (6–12 h); (iii) middle phase (24–72 h), and (iv) the late phase (termination). The immediate early phase, called also the initiation or priming phase, starts 10 min after partial liver removal and is necessary for full response of regenerating hepatocytes to the growth factors involved in regulation of their proliferation [18, 19].

In the adrenal gland the initial or early phase of regeneration is very long (when compared to the liver) and this process is disturbed by the presence of central clot, which becomes an inflammatory site [20, 21]. And, in fact, present studies on regenerating adrenals have revealed a rapid elevation of expression of genes involved in response to wounding, defense response, and in immunological processes. Factors encoded by these genes obscure possible priming effects of various cytokines on initiation of adrenal regeneration. Therefore, in the present study we focused our attention on expression profile of selected genes involved in control of adrenocortical cell proliferation (activators and inhibitors), receptors of growth factors that stimulate adrenocortical proliferation, and transcription factors of known cell cycle regulatory function. Expression profiles of these genes, either up- or downregulated, form a specific pattern related to the duration of experiment and expand our knowledge on control of proliferation of adrenocortical cells in enucleation-evoked regeneration of the rat adrenal cortex.

## 2. Materials and Methods

### 2.1. Animals, Reagents, and Experimental Design

Female Wistar rats (final body weight 120–150 g) from the Laboratory Animal Breeding Center, Department of Toxicology, Poznan University of Medical Sciences, were used. The animals were maintained under standardized conditions of light (14:10 h light-dark cycle, illumination onset at 06.00 a.m.), at 23°C, with free access to standard pellets and tap water. The study protocol was approved by the Local Ethics Committee for Animal Studies (license 19/2011). If not otherwise stated, all reagents were obtained from Sigma-Aldrich (St. Louis, MO, USA) or from Avantor Performance Materials Poland S.A. (Gliwice, Poland).

Adrenal enucleation was performed according to the classic method [22]. Under standard ketamine and xylazine anaesthesia, via dorsal approach, both adrenal glands were enucleated. The procedure was similar to that described earlier [9]. After enucleation, the animals were given 0.9% sodium chloride as drinking water for 3 days, to compensate for their initial incapacity to produce aldosterone. Enucleated rats (4-5 per group) were sacrificed after 1, 2, 3, 5, 8, and 15 postoperative days. Immediately after decapitation adrenal glands were removed and preserved in RNAlater at −20°C. Some adrenals were fixed in Bouins' solution (for immunohistochemistry). Our preliminary data revealed that in adrenals of sham operated rats expression levels of studied genes were similar at days 1 and 15 after surgery; therefore, in the present study the control group combines sham operated rats 24 h after surgery.

### 2.2. RNA Isolation

Expression studies were performed on adrenals of 3 randomly chosen rats from each experimental day. From regenerating and control glands total RNA was extracted using TRI Reagent (Sigma) and purified on columns (RNeasy Mini Kit, Qiagen). The amount of total mRNA was determined by optical density at 260 nm and its purity was estimated by 260/280 nm absorption ratio (higher than 1.8) (NanoDrop spectrophotometer, Thermo Scientific). The applied methods were described earlier [23, 24].

### 2.3. Reverse Transcription

Reverse transcription was performed using AMV reverse transcriptase (Promega) with Oligo dT (PE Biosystems, Warrington, UK) as primers in the temperature of 42°C for 60 min (thermocycler UNO II, Biometra). The primers used were designed by Primer 3 software (Whitehead Institute for Biomedical Research, Cambridge, MA) (Tables 1 and 2). The primers were purchased from the Laboratory of DNA Sequencing and Oligonucleotide Synthesis, Institute of Biochemistry and Biophysics, Polish Academy of Sciences, Warsaw.

### 2.4. Microarray RNA Analysis

Affymetrix Rat Gene 1.1 ST Array method was used as described earlier [9]. Shortly, isolated from regenerated rat adrenal gland total RNA (100 ng) was subjected to two rounds sense cDNA amplification (Ambion WT Expression Kit). The obtained cDNA was used for biotin labelling and fragmentation by Affymetrix GeneChip WT Terminal Labeling and Hybridization (Affymetrix). Microarray results were analyzed using Bioconductor package of R language, based on the Gene Ontology database.

**Figure 1:**
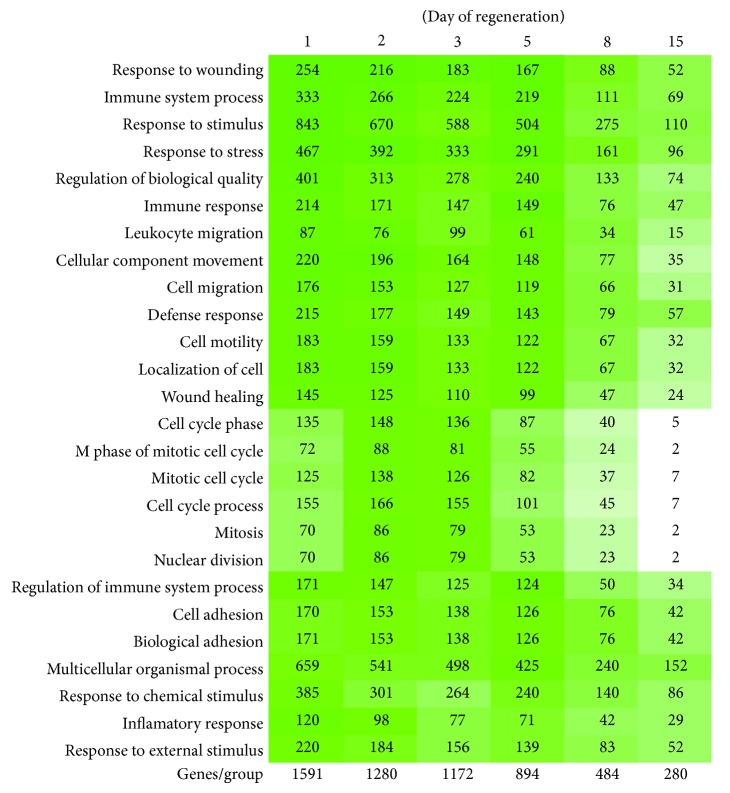
Affymetrix Rat Gene 1.1 ST Array. Ontology analyses of up-/downregulated genes (fold >2, *P* < 0.05) using GeneAnswer (part of the R project) based on Gene Ontology database. On the left side functional categories were placed, on the bottom of the graph—total number of genes up-/downregulated during adrenal regeneration. Genes with changed expression (fold >2, *P* < 0.05) compared to control were grouped in functional categories. In each category hypergeometric statistical test was performed. Green color—statistically significant changes of expression (*P* < 0.05). If *P* value is lower, green color is more intense. It should be emphasized that GO database is composed of some general as well as specific categories with similar meanings and therefore a single gene may be mapped to several GO terms and may be counted more than ones.

The first step of microarray analysis was the preparation of ontology analysis, which allows for interpretation of the group of genes in relation to their participation in specific biological processes (clustering analysis). Only genes which were found up- or downregulated compared to control (*P* < 0.05, >2 fold) during rat adrenal cortex regeneration were grouped into functional categories. GeneAnswer package (part of the R project) based on Gene Ontology database—biological processes (GO.BP) was used at this stage of the study [25]. In each functional category the hypergeometric statistical test was made. The obtained results were presented in Figure 1.

Next, by using in GO database the “cell cycle” words as a query in description of the regeneration process, genes involved only in cell cycle were analyzed. In entire experiment 106 genes involved in cell cycle regulation were identified as up- or downregulated. Expression levels of these genes were presented as a heat map with the hierarchical clustering (Figure 4). Hierarchical clustering was performed on log2 signal intensity data. These values were resized to Row *Z*-score scale for any single genes (from −3—the lowest expression to +3—the highest expression). The lowest expression value was map to red colour, whereas green colour corresponds to the highest expression. Since GO functional annotations of genes are still in developing stage and are far from complete, we also analysed proliferation-associated genes (by means of QPCR) not selected by GO database, but known as essential for cell proliferation.

### 2.5. QPCR

Expression levels of selected genes (listed in Tables 1 and 2) were also validated by means of QPCR (the Lightcycler 2.0 instrument (ROCHE) with the 4.05 software version). Using the above mentioned primers, SYBR green detection system was applied according to the protocol described earlier [9]. Specificity of reaction products was checked by determination of melting points (0.1°C/s transition rate).

### 2.6. Immunohistochemistry

A standard immunohistochemistry method (using peroxidase) with ABC reaction (avidin biotin complex) was used, as described earlier [9, 10]. Anti-Cyclin B1 antibody (abcam ab 2949) was purchased from Abcam (Cambridge, MA, USA).

### 2.7. Statistics

Where appropriate, the data were expressed as means ± SE, and the statistical significance of the differences between control and experimental groups was estimated using Student's *t*-test. It should be underlined that applied GeneAnswer package (part of the R project) based on Gene Ontology database—biological processes (GO.BP) programme—includes also hypergeometric statistical test.

## 3. Results

### 3.1. Microarray Analysis

Ontology analysis of up- and downregulated genes (expression differences higher than 2-fold; *P* < 0.05) in regenerating adrenals revealed a notable, gradual, and parallel to time elapsed from surgery decreases in the number of such genes (Figure 1). 24 h after enucleation, of 1591 genes 254 genes were involved in response to wounding, 467 genes—in response to stress, 215—in defense response, 176—in cell migration, while 170—in cell adhesion and only 70—in mitosis. 48 h after enucleation, the highest statistically significant changes of expression concerned genes involved in response to wounding (216 genes), cellular migration (153 genes), cell motility (159 genes), wound healing (125 genes), immune system processes (266 genes), and response to stress (392 genes). In day 3 of experiment expression levels of genes involved specifically in proliferation, cell cycle (155 genes) and mitosis were found to demonstrate the highest differences. Of interest was that between days 5–15 of regeneration genes involved in response to wounding, stress, immune response, leukocyte migration, and also cell adhesion still were characterized by the most significant changes of expression.

**Figure 2:**
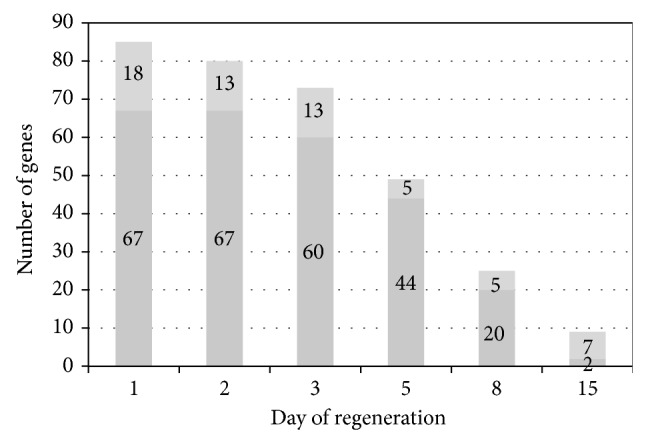
Affymetrix Rat Gene 1.1 ST Array. Number of genes involved in cellular proliferation, which were found to be up-/downregulated during rat adrenal regeneration (*P* < 0.05, fold >2). Dark grey color—increased of expression, light grey—decreased.

**Figure 3:**
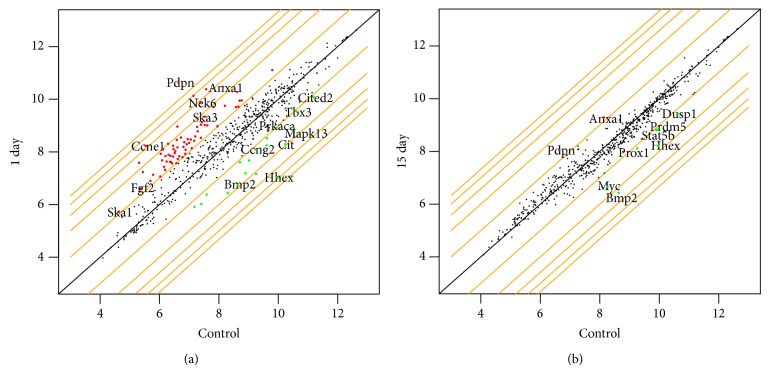
Affymetrix Rat Gene 1.1 ST Array. Expression (log2 signal intensity) of genes involved in cell cycle during enucleation-induced rat adrenocortical regeneration. Scatter plot graphs show data for days 1 and 15 of adrenal regeneration, in relation to control glands. Broken grey lines mark statistically significant changes (fold >2, *P* < 0.05). Dots outside the parallel lines represent transcripts with greater than 2-fold up-/downregulation of expression; upper left part of the graph—increase of expression, lower right part—decrease of expression. At day 1 after enucleation 67 genes were found to be upregulated, for example, Anxa1, Nek6, cyclin E1, and Fgf2, while at day 15 only 2 (Anxa1, Pdpn). At day 1 of regeneration expression of 18 genes decreased (cyclin G2, Mapk13, protein kinase alpha (Prkaca), Bmp2). At day 15 of experiment only 7 genes were downregulated—Bmp2, Prox1, Hhex, Stat5b.

In the next step we analyzed expression of genes involved in cell cycle regulation as well as growth and transcription factor actions on/in adrenocortical cells. The results are presented as a general number of up-/downregulated genes (Figure 2) and as a scatter plot graph (Figure 3). 24 h after enucleation expression of 85 genes was changed (increased/decreased, *P* < 0.05, >2 fold) and 67 of them were found to be upregulated. The group of upregulated genes combined, among other: insulin-like growth factor 1 (Igf1); insulin induced gene 1 (Insig1); Ccne1; Nek6; fibroblast growth factor 2 (Fgf2); UDP glucuronosyltransferase 1 family, polypeptide A5 (Ugt5a1); annexin 1 (Anxa1). On the other hand, at the same time-point of the experiment, expression of 18 genes was downregulated, including cyclin G2 (Ccng2); mitogen activated protein kinase 13 (Mapk13); protein kinase alpha (Prkaca) and bone morphogenetic protein 2 (Bmp2). In contrast, at day 15 after enucleation expression of only 2 genes was upregulated (Anxa1 and podoplanin—Pdpn) and expression of 7 genes was downregulated, including genes of myelocytomatosis oncogene (Myc); Bmp2; transcription factors Prox1 and Hhex; dual specificity phosphatase 1 (Dusp1); signal transducer and activator of transcription 5B (Stat5b) and protein Prdm.

**Figure 4:**
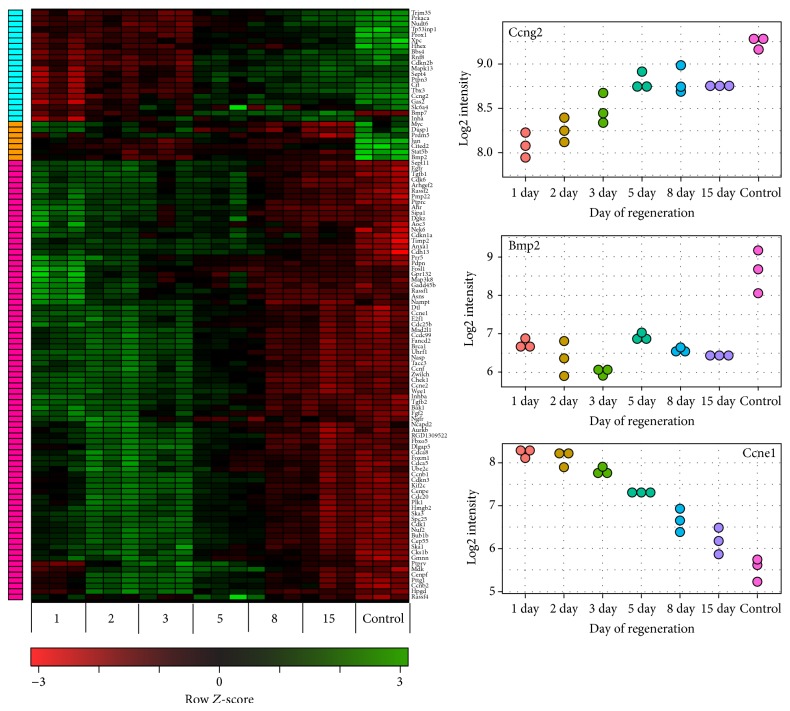
Heat map representation of microarray analysis for the 106 transcripts of genes involved in cell cycle, with the highest differential expression in regenerating adrenals (versus control glands), independent of the day of change (days 1, 2, 3, 5, 8, and 15 of experiment). Signal intensity acquired from microarray analysis is represented by colors (see color key). Green color—increase of expression, red—decrease. Studied genes were clustered by means of hierarchical clustering algorithm. The following clusters were obtained: 20 genes with the highest expression between days 5 and 15 of regeneration (pink); 7 genes with the highest expression in control glands (orange); 79 genes with the highest expression at days 1 to 5 of experiment (blue). C—control animals. Dot-plot graphs present profile expression of 3 genes (Ccng2, Bmp2, and Ccne1) which are representatives for each heat map cluster.

Subsequently expression levels of up-/downregulated genes were presented as a heat map (Figure 4). The studied genes were clustered by means of a hierarchical clustering algorithm and the following clusters were obtained: (1) composed of 20 genes with increased expression between days 5 and 15 of regeneration (pink part); (2) composed of 7 genes only with the highest expression in control glands (orange section); (3) combining 79 genes with the highest expression at days 1 to 5 of the experiment (blue group). An arbitrary signal intensity, acquired from microarray analysis, was represented by colours. As an example of the obtained clusters, expression profiles of 3 genes were shown in form of dot-plot graphs, including genes of cyclin G2, the expression of which increased during adrenal regeneration and at day 15 it was the highest; Bmp2 with the highest expression in control gland and cyclin E1—with the highest expression at the beginning of the regeneration process (Figure 4).

### 3.2. QPCR

**Figure 5:**
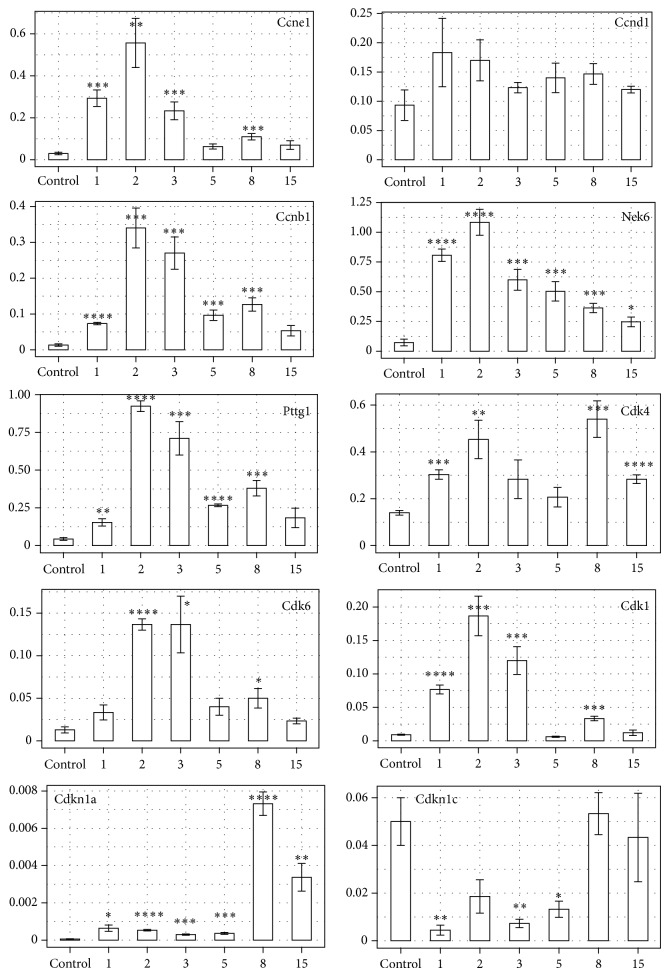
QPCR assay of Ccne1, Ccnd1, Ccnb1, Nek6, Pttg1, Cdk4, Cdk6, Cdk1, Cdkn1a, and Cdkn1c mRNA expression in the regenerating rat adrenal cortex compared to control adrenals. Bars represent mean ± SEM (*n* = 3). All samples were amplified in triplicate, and HPRT gene expression was used as reference to normalize data. Statistically significant differences in relation to control group:  ^*^ *P* < 0.05;  ^**^ *P* < 0.02;  ^***^ *P* < 0.01;  ^****^ *P* < 0.001.

**Figure 6:**
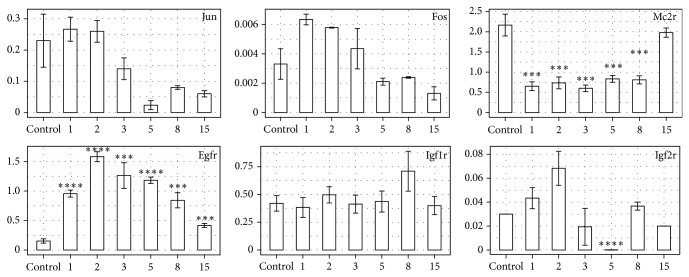
QPCR assay of Junb, Fos, Egfr, Igf1r, Igf2r, and Mc2r mRNA expression in the regenerating rat adrenal cortex compared to control adrenals. Bars represent mean ± SEM (*n* = 3). All samples were amplified in triplicate, and HPRT gene expression was used as reference to normalize data. Statistically significant differences in relation to control group:  ^*^ *P* < 0.05;  ^**^ *P* < 0.02;  ^***^ *P* < 0.01;  ^****^ *P* < 0.001.

The data obtained by means of RNA microarray method were validated by QPCR for selected genes involved in cellular proliferation, the expression of which was most pronounced compared to the remaining genes (Figures 5 and 6). As demonstrated in Figure 5, expression manifested by most of the examined genes, especially stimulators of proliferation, was significantly higher during rat adrenal regeneration when compared to the control. Ccne1, Ccnb1, Pttg1, and Nek6 mRNAs were strongly upregulated, while expression of Ccnd1 mRNA during the examined period was comparable to the control. Furthermore, expression of cyclin-dependent kinases (Cdk4, Cdk6, and Cdk1) was also significantly higher than in the control in almost every day of regeneration. Only at day 5 of regeneration, expression of Cdk4, Cdk6, and Cdk1 mRNA was lower and comparable to the control. Expression profile of Cdkn1a and Cdkn1c mRNA was also analyzed and two distinct profiles were observed. One involved a significant upregulation of Cdkn1a gene (p21) during regeneration, especially at day 8 after enucleation, the second represented a pronounced downregulation of Cdkn1c mRNA (p57) at first 5 days of regeneration with subsequent return to control level.

Expression profiles of genes not selected by GO database, but of directly or indirectly known stimulatory effect on proliferation of adrenocortical cells, were also examined (Figure 6). Expression of epidermal growth factor receptor gene was significantly upregulated at every day of adrenal regeneration, compared to control. Whereas expression of Igf1r gene during regeneration process was comparable to control, only in day 8 expression of the gene proved to be increased. In turn, expression of Igf2r gene was significantly increased in day 2, whereas in day 5 after enucleation it was abruptly decreased, as compared to the control. Expression of Mc2r gene was found to be strongly decreased till day 8 of regeneration, but afterwards it was increased. We also studied expression profile of two transcription factors known to be involved in the regulation of the cell cycle. Expression profile of Junb gene was comparable to control at the beginning of regeneration, while at days 5, 8, and 15 of regeneration it decreased and remained lower than in the control. On the other hand, expression of Fos gene increased in days 1 and 2 after surgery and subsequently decreased. Changes of expression profile of Junb, Igf1r, and Fos were not statistically significant.

**Figure 7:**
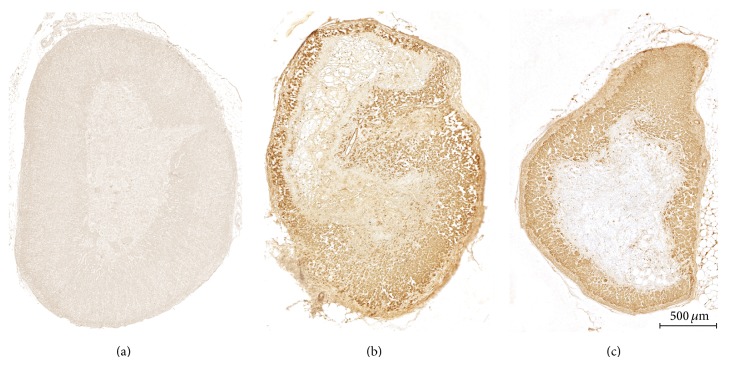
Cyclin B1-like immunoreactivity in regenerating rat adrenal cortex. Cytoplasmic staining of adrenocortical cells in regenerating glands: (b) day 3 and (c) day 5 of regeneration. (a) Adrenal of sham operated rat (counterstained with haematoxylin). Magnification defined by the scale bar.

In enucleation-induced adrenal regeneration immunohistochemistry revealed intense Cyclin B1-like immunoreactivity in the cytoplasm of all adrenocortical cells, while in control gland no such reaction has been observed (Figure 7).

## 4. Discussion

It is well known that under both experimental (for example enucleation or transplantation of the gland) and clinical (mainly transplantation) conditions adrenocortical cells have great ability to regenerate. In the rat an almost complete removal of the cortex and medulla (enucleation) of the gland triggers a rapid regeneration of the cortex and very slow regeneration of adrenal chromaffin cells [22, 26]. Adrenocortical cells regenerate immediately after enucleation from subcapsular progenitor/stem cells as well as zona glomerulosa cells left after enucleation surgery [14, 15, 27]. Within 8 days after surgery, adrenal zona fasciculata is well restored while reconstruction of the zona glomerulosa remains incomplete before day 30 of experiment [16]. Initial steps of adrenal regeneration are burdened by massive bleeding from the capsule and formation of central clot in remnants of the gland, surrounded by connective tissue. On the other hand, partial hepatectomy-induced liver regeneration is affected neither by bleeding nor clot formation. Therefore, data obtained in this model may serve as guidance in search for similar changes in regenerating adrenals. However, it should be emphasized that adrenals regenerate from stem and progenitor as well as undifferentiated cells while livers regenerate primarily from mature (highly differentiated) cells and probably from some liver stem cells [5, 7, 19, 28].

In regenerating liver, the priming phase starts 10 min after surgery and is indispensable for full response of regenerating hepatocytes to the growth factors involved in regulation of their proliferation [18, 19].* In vitro* priming requires cytokines (among others TNF, IL-6), which stimulate expression of such transcription factors as NFKB, STAT3, AP-1, and CIEBPP [18]. Their concerted action prepares regenerating hepatocytes to respond to growth factors (for example HGF—hepatocyte growth factor; TGF*α*—transforming growth factor alpha; EGF—epidermal growth factor).

It is well documented that growth of the regenerating adrenal gland and restoration of its function are regulated by different factors, released by the vascular system cells as well as nerve cells and the immune system cells [9, 29–33]. Our earlier studies revealed that in enucleation-induced regenerating rat adrenals over 2,000 genes were strongly up- or downregulated [9]. At the beginning of the regeneration (days 1–8 after enucleation) expression of genes involved in processes like inflammation, response to wounding, response to stress, immune system processes, cell adhesion, cell migration, blot clot formation, and angiogenesis was significantly increased. Likewise, in regenerating liver genes involved in acute-phase and defense responses were rapidly elevated in early phases while those regulating cell proliferation and DNA replication were significantly upexpressed in the middle phase [17].

In the present study we have focused our attention on expression profiles of genes involved in control of adrenocortical cell proliferation in regenerating rat adrenal gland. In this regard in regenerating adrenals we have identified over 100 up- or downregulated genes involved in adrenocortical cell proliferation and changes in their expression were most pronounced at days 2 and 3 after enucleation. Number of genes up/downregulated involved in cell cycle regulation has decreased during the examined period of rat adrenal regeneration. At day 1 after enucleation, 85 genes have been up-/downregulated, while at day 15 of the process only 7 such genes have been found, among them genes directly involved in cell cycle regulation, for example, cyclins (cyclin E1, E2, and B1), cyclin-dependent kinases (Cdk6 and Cdk1)—enzymes that regulate transcription, mRNA processing and progression of cell cycle, also enzymes involved in separation of sister chromatids during anaphase, centromere proteins (Cenpe and Cenpf), and kinetochore proteins (Ska3). They have included also genes indirectly involved in proliferation, like transcription factors (Jun), growth factors, and its receptors (Igf1 and Egfr) and intracellular metabolic pathways proteins or their activators (Mapk13, Map3k8—mitogen-activated protein kinase kinase kinase 8). Expression of most of the above mentioned genes has increased in the beginning of the rat adrenal regeneration, especially between days 1 and 5. In this regard it should be mentioned that ^3^H-thymidine histoautoradiography demonstrated the highest mitotic activity of regenerating adrenal cortex at day 5 of experiment [2]. Furthermore, our data have indicated that at day 15 of experiment expression level of most of the genes involved in control of adrenocortical proliferation has been comparable to control.

Numerous regulatory proteins are involved in control of cell cycle and some of the most important are cyclins, cyclin-dependent kinases, and their inhibitors. Together these proteins regulate progression of cell cycle. On different stages of cell cycle expression of different cyclins, Cdks and cell cycle inhibitors (CKI) must appear. For example, high level of cyclins E1 and D1 is essential for a cell to cross first restriction point (R) at the end of G1 phase. Our results have indicated high expression levels of Ccne1 mRNA at the beginning of regeneration (days 1–3), while expression of Ccnd1 has not changed during adrenal regeneration. Furthermore, high concentration Cdk4 and Cdk6 protein in cell is required at this time of cell cycle as well. As demonstrated, expression level of Cdk4 and Cdk6 mRNAs has been significantly upregulated almost every day of regeneration while expression levels of cell cycle inhibitors have been lowered. Fraticelli and coworkers demonstrated earlier that mRNA concentration of p57 protein was low at the beginning of regeneration, but at day 5 it increased and at day 7 it was higher than in control [34]. Our results have confirmed these earlier studies and have indicated that expression of p57 has been much lower than in the control in the beginning of regeneration (days 1–5). On the other hand, p21 mRNA (also Cdks inhibitor) has been strongly upregulated almost every examined day of regeneration. p21 protein functions as a regulator of cell cycle progression at G1 and S phases and is considered to be a potent regulator of hepatocyte proliferation during liver regeneration [19]. High expression of p21 gene inhibits the cell cycle in G1 phase and mediate replicative senescence. Adrenocortical cells cease to divide and are directed into G0 phase and subsequently probably differentiate into zona fasciculata cells. This assumption may be confirmed by our earlier findings demonstrating that at day 5 of adrenal regeneration, expression of Cyp11b1 reached level comparable to control [10].

Our QPCR results have revealed also a notable upregulation of expression of cyclin B1 and Cdk1 mRNA. As known, high expression of both genes is necessary for cell to cross the checkpoint at the end of G2 stage of interphase.

Progression of cell cycle is regulated also by growth arrest genes, for example, Gadd45 (growth arrest and DNA-damage-inducible, alpha; NM_024127.2) which delays G2-M transition by induction of DNA repair. In our hands this gene has been upregulated 24 hours after enucleation surgery (microarray analysis). Likewise, this gene was found to be upregulated during priming phase of mice liver regeneration [19].

There are several important mitosis restriction points, for example, formation of metaphase in equatorial plane or separation of sister chromatids during anaphase, in which Nek6 and Pttg1 genes play an important role. Nek6 is known as serine/threonine protein kinase, involved in metaphase stage of mitosis and its inhibition lead to apoptosis, while Pttg1, a substrate of anaphase-promoting complex (APC), participates in separation of chromatids into opposite poles of the cell. Using microarray and QPCR methods we have demonstrated that expression of both genes was significantly upregulated in every day of adrenal regeneration, with peak at day 2 after enucleation. Scanty information is available on Nek6 and Pttg1 functions in adrenocortical cells. However, it has been known that these genes are necessary for mitotic spindle formation and functioning during mitosis. NIMA kinase 6 (Nek6) act probably with Cdk1 and contributes to separation of centrosomes [35, 36]. On the other hand, earlier data indicated that Pttg1 enhanced proliferation of keratinocytes [37].

Growth of adrenal gland* in vivo* is regulated also by numerous growth factors and their receptors, for example, basic fibroblast growth factor (bFgf), insulin-like growth factors (Igf1 and Igf2), and epidermal growth factor (Egf) [38–42]. Igf1 mRNA levels are very high in the zona fasciculata cells and Igf1 is known to stimulate proliferation and steroidogenesis in human, rat, and bovine adrenocortical cells [39, 43, 44]. Furthermore, expression of Igf1 receptors is upregulated by ACTH and its elevated expression in regenerating adrenals, as observed in our study, may be caused by compensatory hypersecretion of ACTH [45, 46]. Igf2 is also recognized as a growth factor exerting stimulating effect on fetal adrenal gland [44]. However, as demonstrated by our microarray data, significant upregulation of Igf1 gene was seen at the beginning of rat adrenal regeneration (especially days 1–5) while expression levels of Igf2, Igf1r, and Igf2r genes remains rather unchanged (data not shown). Earlier studies of Gospodarowicz et al. [47] identified bFgf as a mitogenic and migratory agent, regulating bovine and rat adrenocortical cells. bFgf is also known as a growth factor that may mediate proliferation of adrenocortical cells during rat compensatory adrenal growth and adrenal regeneration [38, 42]. Our results have confirmed these earlier studies, since expression of bFgf gene has been increased at the initial period of adrenal regeneration.

Another growth factor—Egf—stimulates adrenocortical growth and functional maturation and this action is connected with induction of expression of 11beta-hydroxylase (Cyp11b) and 3beta-hydroxysteroid dehydrogenase (3bHsd) in the fetal rhesus monkey [48]. In our experiments expression of Egf gene has not significantly changed during adrenal regeneration (microarray data, not shown). On the contrary, as demonstrated by QPCR analysis, in regenerating adrenals expression of Egfr gene has been significantly upregulated during the entire experiment (till day 15). This finding is very interesting, since Egfr and its ligands (Tgfa—tumor necrosis factor alfa and Hbegf—heparin-binding Egf-like growth factor) play an important mitogenic role in primary culture of hepatocytes as well as during partial hepatectomy induced liver regeneration [49, 50]. This stimulating effect of Hbegf on cell cycle progression was earlier observed by other groups [51, 52]. Another Egfr ligand, Tgfa also stimulates cell proliferation and motility and blood vessel formation. This ligand is also well known as a priming factor during liver regeneration [18]. Our microarray analysis has revealed significantly higher expression of Tgfa gene only 24 hours after enucleation surgery and this finding suggests that its role in adrenal regeneration may be analogous to that observed in regenerating liver.

It is well known that proliferation of adrenocortical cells is controlled principally by proopiomelanocortin-derived peptides (POMC-peptides), especially N-terminal POMC-peptides [12, 53]. Adrenal enucleation-evoked rapid decrease in blood corticosterone concentrations results in a rapid compensatory hypersecretion of pituitary ACTH and other N-POMC-derived peptides [54].* In vivo* stimulating effect of ACTH on adrenocortical cell proliferation is secondary to its effect on the cell size [1, 55]. Of interest are observations that in rats with regenerating adrenals, despite elevated blood ACTH concentrations, ACTH administration attenuates proliferative activity of adrenocortical cells, although such treatment increases the ratio of DNA/RNA in studied cells [3, 55]. In this regard in regenerating adrenals we have followed the pattern of expression of Mc2r gene (ACTH receptor). Unexpectedly, during the first 8 days of experiment Mc2r RNA levels have been notably lower than in control glands and only at day 15 they have exceeded values seen in sham operated rats. These data suggest that enucleation-induced rat adrenal cortex regeneration does not require elevated expression of Mc2r. On the other hand it is well known that ACTH-receptor gene is unique in that it is upregulated by its own ligand. Thus, these puzzling changes observed in present study remain to be elucidated in future. On the other hand, another N-POMC peptide was earlier found to exert potent mitogenic activity on adrenocortical cells [56]. The peptide is cut off the N-POMC precursor by specific adrenal protease (Asp, Tmprss11d, NM_001033652.1) which is expressed in the outer part of adrenal cortex. Our microarray analysis has revealed no significant changes in expression profile of Asp gene during adrenocortical regeneration (data not shown).

Under physiological conditions cellular proliferation is stimulated by protooncogenes such as Jun, Junb, and Fos, which then form (Jun and Fos) transcriptional factor AP-1 [57]. Jun and Fos protooncogenes were identified as important transcription factors activated very early (10 minutes) after partial hepatectomy [19]. In adrenocortical cells Fos gene is induced by both ACTH and Fgf2 [58–60]. In our experiments in the beginning of regeneration (days 1-2) only Fos gene has been found to be upregulated, while between days 5 and 15 after enucleation a lowered expression of both genes has been found.

Thus, the performed studies have revealed that enucleation-induced adrenal regeneration resulted in rapid upregulation of genes involved in response to wounding, defense, and stress responses and in immunological processes. At 24 h after surgery expression levels of immediate-early transcription factors have been notably elevated. The most significant changes in expression levels of genes involved in cell cycle regulation have occurred at days 2 and 3 of adrenal regeneration. Despite notable differences in mechanisms of adrenal and liver regeneration, in regenerating adrenals we have identified genes, the expression of which is well recognized in regenerating liver, for example, Tgfa or Fos, both belonging to primers of liver regeneration. In view of these findings it seems legitimate to suggest that, in the rat, the general model of liver and adrenal regeneration demonstrate some degree of similarity.

## Figures and Tables

**Table 1:** List of genes whose profile expression during enucleation-induced rat adrenal regeneration was validated using QPCR method.

Genbank accession number	Gene symbol	Name	Function
NM_001100821.1	Ccne1	cyclin E1	Regulators of Cdk kinases, stimulation of cell growth
NM_171992	Ccnd1	cyclin D1	
NM_171991.2	Ccnb1	cyclin B1	

NM_022391.2	Pttg1	Pituitary tumor-transforming 1	A homolog of yeast securin proteins and is an anaphase-promoting complex substrate

NM_001277232.1	Nek6	NIMA-related kinase 6	A serine/threonine protein kinase during mitosis

NM_053593	Cdk4	Cyclin-dependent kinase 4	Enzymes that regulate transcription, mRNA processing, and progression of cell cycle
NM_001191861.1	Cdk6	Cyclin-dependent kinase 6	
NM_019296.1	Cdk1	Cyclin-dependent kinase 1	

NM_080782	Cdkn1a	Cyclin-dependent kinase inhibitor 1A; p57 protein	Cell cycle inhibitors (Cdks inhibitors)
NM_001033757	Cdkn1c	Cyclin-dependent kinase inhibitor 1C; p21 protein	

NM_031507.1	Egfr	Epithelial growth factor receptor	Promotes cell proliferation and differentiation; mediates GPCR regulated induction of protein synthesis

NM_052807.2	Igf1r	Insulin-like growth factor receptor 1	Receptor for Igf-1; involved in induction of cell cycle progression and survival in many cell types

NM_012756.1	Igf2r	Insulin-like growth factor receptor 2	Mediates internalization of insulin-like growth factor II; plays a role in lysosomal enzyme transport

NM_001100491.1	Mc2r	Melanocortin 2 receptor	ACTH receptor, may mediate peripheral stress responses

NM_022197.2	Fos	FBJ osteosarcoma oncogene; c-fos	An immediate early gene encoding a nuclear protein involved in signal transduction

NM_021836.2	Junb	Jun B proto-oncogene	Transcription factor, involved in transcriptional regulation

**Table 2:** Conventional RT-PCR and QPCR analyses of Ccne1—cyclin E, Ccnd1—cyclin D1, Ccnb1—cyclin B, Nek6—NIMA- (never in mitosis gene a-) related kinase 6, Pttg1—pituitary tumor-transforming 1, Cdk4—cyklin-dependent kinase 4, Cdk6—cyklin-dependent kinase 6, Cdk1—cyklin-dependent kinase 1, Cdkn1a—cyclin-dependent kinase inhibitor 1A, Cdkn1c—cyclin-dependent kinase inhibitor 1C, Egfr—epithelial growth factor receptor, Igf1r and Igf2r—insulin growth factor receptors 1 and 2, Mc2r—ACTH receptor, Fos—FBJ osteosarcoma oncogene, transcription factor and Junb—jun B proto-oncogene, transcription factor. Oligonucleotide sequences for sense (S) and antisense (A) primers are shown. Hprt (hypoxanthine phosphorybosyl transferase) was the reference gene.

cDNA	Genbank accession number	Primer	Primer sequence (5′-3′)	Position	PCR product size (bp)
Ccne1	NM_001100821.1	S	AAGACTGTGAAAAGCCAGGA	320–339	176
		A	AAGACGGGAAGTGGGGAGG	477–495	

Ccnd1	NM_171992	S	AACAAGCAGATCATCCGCAAA	645–666	213
		A	GAGGCAGTCCGGGTCACA	840–858	

Ccnb1	NM_171991.2	S	TTAAAGCCCTACCAAAACC	313–331	207
		A	CAAGAATCACATCGGAGAA	501–519	

Nek6	NM_001277232.1	S	CAGCCCAGCCACATGCCTC	32–50	151
		A	CTCGGCCAATCTTCTTCTC	164–182	

Pttg1	NM_022391.2	S	GTCTCTCCCCTCAGTAATCCA	258–278	171
		A	CACCGAACACTTTGCCGACT	409–428	

Cdk4	NM_053593	S	CAAGTAATGGGACAGTTAAG	631–651	208
		A	GAGTTCCCACAGAAGAGAG	820–839	

Cdk6	NM_001191861.1	S	AATAAAACTGGCTGACTTCGG	474–494	205
		A	ATCCACGTCTGAACTTCC	661–678	

Cdk1	NM_019296.1	S	ACAGAGAGGGTCCGTTGT	67–85	175
		A	CGTACTGGGCACTCCTTCTT	222–242	

Cdkn1a	NM_080782	S	CTTGTCGCTGTCTTGCACT	377–395	241
		A	CACTGAATGAAGGCTAAGG	599–618	

Cdkn1c	NM_001033757	S	GCCTCTCTCGGGGATTCCA	1121–1140	240
		A	TCTAAACTAACTCATCGCAGAC	1339–1361	

Egfr	NM_031507.1	S	ATTGCCCTGAACACCGTGGA	424–443	176
		A	CGCACAGCACCGATCAGAA	581–599	

Igf1r	NM_052807.2	S	GACAGGAGTACAGGAAGTATGG	2693–2714	197
		A	AATCAGCAGGATGGCAACC	2871–2889	

Igf2r	NM_012756.1	S	CATCTCTGTTCATCAATGTG	676–695	164
		A	CCTGTCCTTGCTCAATAG	822–839	

Mc2r	NM_001100491.1	S	GGACAAGGGGGGAGGCAGA	100–119	201
		A	TGGCACAACTACATCAGGAC	281–301	

Fos	NM_022197.2	S	TTTCAACGCGGACTACGAG	167–185	164
		A	AGTTGGCACTAGAGACGGAC	311–330	

Junb	NM_021835.3	S	AATGGAACAGCCTTTCTATCA	282–302	99
		A	GGTTTCAGGAGTTTGTAGTC	361–380	

Hprt	NM_012583	S	CAGTCAACGGGGGACATAAAAG	391–412	146
		A	ATTTTGGGGCTGTACTGCTTGA	515–536	
